# Effect of a four-week oral Phe administration on neural activation and cerebral blood flow in adults with early-treated phenylketonuria

**DOI:** 10.1016/j.nicl.2024.103654

**Published:** 2024-08-14

**Authors:** Stephanie Maissen-Abgottspon, Leonie Steiner, Raphaela Muri, Dilmini Wijesinghe, Kay Jann, Yosuke Morishima, Michel Hochuli, Roland Kreis, Roman Trepp, Regula Everts

**Affiliations:** a Department of Diabetes, Endocrinology, Nutritional Medicine and Metabolism, Inselspital, Bern University Hospital and University of Bern, Switzerland; b Support Center for Advanced Neuroimaging (SCAN), University Institute for Diagnostic and Interventional Neuroradiology, Inselspital, Bern University Hospital and University of Bern, Switzerland; c Translational Imaging Center (TIC), Swiss Institute for Translational and Entrepreneurial Medicine, Bern, Switzerland; d Laboratory of Functional MRI Technology (LOFT), Stevens Neuroimaging and Informatics Institute, Keck School of Medicine, University of Southern California, USA; e Translational Research Center, University Hospital of Psychiatry and Psychotherapy, University of Bern, Switzerland; f Magnetic Resonance Methodology, Institute of Diagnostic and Interventional Neuroradiology, Inselspital, Bern University Hospital and University of Bern, Switzerland; g Neuropediatrics, Development and Rehabilitation, University Children’s Hospital, Inselspital, Bern, Switzerland

**Keywords:** Phenylketonuria, functional MRI, Cerebral blood flow, Cognition, Randomized control trial

## Abstract

**Background:**

Phenylketonuria (PKU) is a rare inborn error of metabolism characterized by impaired catabolism of the amino acid phenylalanine (Phe) into tyrosine. Cross-sectional studies suggest slight alterations in cognitive performance and neural activation in adults with early-treated PKU. The influence of high Phe levels on brain function in adulthood, however, remains insufficiently studied. Therefore, we aimed to explore the effect of a four-week period of oral Phe administration − simulating a controlled discontinuation of Phe restriction and raising Phe to an off-diet scenario − on working memory-related neural activation and cerebral blood flow (CBF).

**Methods:**

We conducted a randomized, placebo-controlled, double-blind, crossover, non-inferiority trial to assess the effect of a high Phe load on working memory-related neural activation and CBF in early-treated adults with classical PKU. Twenty-seven patients with early-treated classical PKU were included and underwent functional magnetic resonance imaging (fMRI) of the working memory network and arterial spin labeling (ASL) MRI to assess CBF before and after a four-week intervention with Phe and placebo. At each of the four study visits, fMRI working memory task performance (reaction time and accuracy) and plasma Phe, tyrosine, and tryptophan levels were obtained. Additionally, cerebral Phe was determined by ^1^H-MR spectroscopy.

**Results:**

Plasma Phe and cerebral Phe were significantly increased after the Phe intervention. However, no significant effect of Phe compared to placebo was found on neural activation and CBF. Regarding fMRI task performance, a significant impact of the Phe intervention on 1-back reaction time was observed with slower reaction times following the Phe intervention, whereas 3-back reaction time and accuracy did not differ following the Phe intervention compared to the placebo intervention.

**Conclusion:**

Results from this present trial simulating a four-week discontinuation of the Phe-restricted diet showed that a high Phe load did not uniformly affect neural markers and cognition in a statistically significant manner. These results further contribute to the discussion on safe Phe levels during adulthood and suggest that a four-week discontinuation of Phe-restricted diet does not demonstrate significant changes in brain function.

## Highlights

• We simulated a temporary discontinuation of the Phe-restricted diet in adults with PKU.
• No effect of high Phe was found for neural activation and cerebral blood flow.
• Reaction time but not accuracy was worse after Phe compared to placebo.
• Differential effects of high Phe on neural markers and cognition are discussed.

## 1 Introduction

Phenylketonuria (PKU) is a rare inborn error of metabolism characterized by high phenylalanine (Phe) concentrations in the blood and brain due to impaired activity of the enzyme phenylalanine hydroxylase (Blau et al., 2010). Impaired metabolism of Phe to tyrosine results in the accumulation of Phe and has detrimental effects on the developing brain. To avoid cognitive and neurological long-term sequelae, such as severe intellectual impairments, seizures, or motor deficits, a Phe-restricted diet combined with a Phe-free amino acid supplementation should be maintained throughout childhood (Blau et al., 2010). The introduction of newborn screening with early-initiated treatment of PKU was a big success in medicine, allowing patients to develop normally and preventing cognitive and neurological problems (Berry et al., 2013).

Two pharmacological treatments have been approved for the treatment of PKU – sapropterin dihydrochloride (BH_4_) and pegvaliase – however, a considerable number of adults with classical PKU do not respond to these treatments or experience side effects, leaving a Phe-restricted diet the treatment of choice (Fiege and Blau, 2007, Hausmann et al., 2019, Sacharow et al., 2020). Adherence to dietary protein restriction is associated with challenges, and many adults report higher Phe levels than recommended (Ford et al., 2018, MacDonald et al., 2010, Ahring et al., 2011). Also, there is no consensus on how strict the treatment should be in adulthood, except before and during pregnancy. The European guidelines suggest a Phe concentration of <600 µmol/L, whereas the American guidelines recommend a Phe concentration of <360 µmol/L (Van Wegberg et al., 2017, Burgard et al., 2017). Consequently, it has been proposed to follow a more individual approach regarding target Phe levels for adults rather than a rigid adherence to the guidelines (Burgard et al., 2017, Lachmann and Langeveld, 2024).

Task-based functional magnetic resonance imaging (fMRI) offers a unique opportunity to study neural networks associated with the execution of a particular task (Poldrack and Farah, 2015). Cross-sectional studies show that neural activation and connectivity within the fronto-parietal working memory network are slightly altered in adolescents and adults with PKU compared to controls (Christ et al., 2010, Abgottspon et al., 2022). Investigating neural activation associated with inhibition, another aspect of executive functions next to working memory (Miyake et al., 2000), no or only subtle activation differences were observed in adults with early-treated classical PKU compared to healthy controls (Sundermann et al., 2020). Only a limited number of interventional fMRI studies have been conducted in PKU. Sundermann et al. (2011) administered a single oral Phe load in 17 adults with PKU and did not find any changes in neural activation associated with inhibition before and after the Phe load. Controversially, alterations in neural activation in the fronto-parietal working memory network were observed in seven children and adults only after successful implementation of treatment with sapropterin dihydrochloride (Kuvan®, Christ et al., 2013). We showed in a sample of 29 adults with early-treated classical PKU that a four-week period of oral Phe administration did not negatively impact working memory, manual dexterity, mood, and depression (Trepp et al., 2024). However, sustained attention differed following the Phe intervention compared to the placebo intervention, although it is worth mentioning that the significance primarily arose from the improvement observed during the placebo intervention (Trepp et al., 2024). Whether and how a four-week suspension of the Phe-restricted diet affects neural activation in the fronto-parietal working memory network remains to be investigated.

In addition to fMRI, arterial spin labeling (ASL) allows for noninvasive investigation of cerebral perfusion (Haller et al., 2016). This technique uses water as an endogenous tracer, which thus enables the quantification of cerebral blood flow (CBF). ASL serves as a valuable complement to fMRI as it provides a quantitative measure for assessing CBF, while fMRI offers an indirect measure of neural activation through neurovascular coupling (Detre and Wang, 2002). ASL has been employed in various clinical populations, such as dementia or stroke, to examine potential long-term alterations in CBF (Haller et al., 2016, Zaharchuk, 2014). It has further been used to investigate acute changes in CBF during cognitive performance, enabling the detection of regional increases and decreases in CBF (Kim et al., 2006). A limited number of studies investigated cerebral perfusion in early-treated adults with PKU. Our cross-sectional study showed no significant global CBF differences between adults with PKU and controls (Steiner et al., 2024). However, reduced mean CBF was observed in arterial vascular territories of the left middle cerebral artery (MCA) and the posterior cerebral artery (PCA) in patients with PKU, a finding that was unrelated to cognitive performance and metabolic parameters.

Results of the cross-sectional study of the present sample have previously been described (see Abgottspon et al., 2022, Steiner et al., 2024). The aim of the present randomized, placebo-controlled, double-blind, crossover, non-inferiority trial was to assess the effect of a short-term high Phe load − simulating a four-week discontinuation of the Phe-restricted diet − on working memory-related neural activation and resting CBF in adults with early-treated classical PKU. We hypothesized that a high Phe load compared to placebo does not modulate neural activation in the working memory network or CBF. We further aimed to investigate associations between neural markers, cognition, and metabolic parameters after the Phe and placebo intervention.

## 2 Material and methods

Details about the study design, participants, interventions, randomization, and blinding have been published previously (Abgottspon et al., 2022, Trepp et al., 2024, Steiner et al., 2024, Trepp et al., 2020, Muri et al., 2024) and are summarized below.

### 2.1 Study design

We conducted a randomized, placebo-controlled, double-blind, crossover, non-inferiority trial in adults with early-treated PKU to examine the impact of Phe compared to placebo. Patients were randomly allocated to first receive Phe followed by placebo (Phe-placebo) or first receive placebo followed by Phe (placebo-Phe). Both intervention periods lasted four weeks and were separated by a four-week washout phase. Four study visits (T1–T4) before and after each intervention period were performed at the Department of Diabetes, Endocrinology, Nutritional Medicine and Metabolism of the University Hospital in Bern, including a blood sample, a neuropsychological, and neuroimaging assessment. T1 took place on the day the first intervention period began, T2 at the end of the first intervention period, T3 at the start of the second intervention period, and T4 at the end of the second intervention period. Here, we report results from the fMRI addressing functional brain networks involved in working memory and CBF assessed with ASL.

The trial was approved by the local Ethics Committee of Bern (2018-01609), was conducted in accordance with the ethical principles of the Declaration of Helsinki, and was registered on clinicaltrials.gov (NCT03788343). Written informed consent was obtained from all participants.

### 2.2 Participants

Recruitment took place between July 2019 and June 2022, with an interruption from March 2020 to May 2020 due to the COVID-19 pandemic. All patients were recruited via their metabolic specialists in Bern (Switzerland), Zurich (Switzerland), Basel (Switzerland), Lausanne (Switzerland), Hamburg (Germany), Ulm (Germany), and Innsbruck (Austria). All patients were ≥18 years old and diagnosed with classical PKU after a positive newborn screening with an initiation of the Phe-restricted diet within 30 days of life. Patients not following a Phe-restricted diet or with Phe concentrations >1600 µmol/L, treated with sapropterin dihydrochloride (Kuvan®) or pegvaliase (Palynziq®), or displaying conditions interfering with the study protocol or the MRI acquisition (e.g., pregnancy, lactating) were excluded.

### 2.3 Intervention

An oral administration of Phe or placebo was implemented to simulate a controlled discontinuation of the Phe-restricted diet. Depending on the weight and sex of the patient, 1500–3000 mg Phe per day divided into three doses was administered, for details see (Trepp et al., 2024). Patients received an identical number of capsules containing placebo (pregelatinized corn starch, Lycatab C) during the placebo intervention. Phe and placebo capsules were identical in appearance, package, and labeling.

### 2.4 Randomization and masking

Eligible patients were randomly allocated to group 1 (first Phe, then placebo) or group 2 (first placebo, then Phe) using computer-generated central randomization with age, sex, and site of usual medical care (i.e., study center site Bern or elsewhere) as stratification factors. Computer-generated central randomization was performed by an independent statistician and transferred to the Laboratorium Dr. G. Bichsel AG, which produced the Phe and placebo capsules. All study participants and personnel were blinded to the patient’s intervention allocation.

### 2.5 Neuroimaging acquisition and analysis

All patients underwent a task-based fMRI and a resting ASL examination. For details regarding the fMRI and ASL acquisition and analyses, see Abgottspon et al., 2022, Steiner et al., 2024). In short, the neuroimaging acquisition was performed on a 3-Tesla Siemens Magnetom Prisma whole-body scanner (Siemens Erlangen, Germany) equipped with a 64-channel head coil. Functional images were obtained using multi-slice single-shot T2*-weighted echo-planar imaging (TR = 1000 ms, TE = 30 ms, TA = 9:52 min, FA = 80°, FOV = 192 × 192 mm, 48 slices, isotropic voxel resolution = 2 mm^3^). A magnetization-prepared rapid acquisition gradient-echo (MP-RAGE) sequence was used to obtain anatomical images (TR = 1950 ms, TE = 2.26 ms; TI = 900 ms, TA = 4:34 min, FA = 9°, FOV = 256 mm × 256 mm, matrix dimension = 256 × 256, isotropic voxel resolution = 1 mm^3^). CBF was acquired using a pulsed arterial spin labeling (pASL) sequence (TR = 8000 ms, TE = 16.18 ms, TI = 1500 ms, TA = 4.59 min, bolus duration = 800 ms, FA = 180°, FOV = 192 mm × 192 mm, voxel resolution = 1.5 × 1.5 × 3.0 mm, 4 label/control pairs) and an M0 image (TR = 8000 ms, TI = 7000 ms, TA = 2.16 min).

Regarding the fMRI sequence, a visuo-spatial working memory task was implemented with a 1-back condition as baseline and a 3-back condition as working memory condition. The design was adapted from Jaeggi et al. (2010) and presented in a block design with a total of eight blocks (four blocks per condition). Each block lasted 60 s and contained 20 stimuli presented for 500 ms with a 2500 ms inter-stimulus interval. Accuracy and reaction time were collected for each participant and condition. To address motion-related artifacts in the fMRI time-series, we used the Artifact Detection Tools (ART) toolbox (https://www.nitrc.org/projects/artifact_detect). All volumes with a global signal z-threshold >5 and a movement threshold >0.09 (which corresponds to the 97th percentile setting) were identified as outlier volumes, which were then entered as regressors of no interests in the first-level analyses (in addition to the six motion parameters from the rigid body realignment). Participants with more than 15 % of volumes identified as outliers were excluded from the analyses (*n* = 2).

The SPM12 (Wellcome Trust Centre for Neuroimaging, London, UK) software was used for preprocessing and analysis. Preprocessing steps included: (i) realignment and reslicing of all functional images to the mean functional image using a six-parameter rigid body transformation to correct for motion distortion; (ii) coregistration of the mean functional image to the structural image; (iii) segmentation and spatial normalization into the Montreal Neurological Institute (MNI) space with a voxel size of 2 × 2 × 2 mm^3^; (iv) smoothing of functional images with a Gaussian kernel of 8 mm full-width at half-maximum . The general linear model was applied to obtain individual contrast images (“3-back > 1-back”), which were then used for second-level analyses and region-of-interest (ROI) analyses. As we were particularly interested in fronto-parietal working memory activation, we used nine independently defined ROIs based on the Neurosynth database. We searched the term “working memory” and obtained an association map from 1091 studies (threshold z = 3.7). From the 85 identified clusters, we included only those ≥100 voxels, excluding clusters in the cerebellum, resulting in nine ROIs for further analysis. Specifically, the clusters were located in the left and right insula, right inferior frontal gyrus, right middle frontal gyrus, right superior frontal gyrus, left and right inferior parietal gyrus, and two clusters in the left middle frontal gyrus, with the sizes of the ROIs varying between 144 and 2311 voxels (each voxel being 2 × 2 × 2 mm^3^, for details see Abgottspon et al., 2022). This approach ensures our focus on relevant, substantial activations while maintaining specificity to working memory. Parameter estimates (betas) of the contrast 3-back > 1-back were extracted using MarsBar (Brett et al., 2002) and analyzed in SPSS. Furthermore, we calculated the average activation across all nine ROIs to determine the activation for the entire working memory network. This was done by first combining the nine ROIs into a single mask and then extracting the mean beta value from this combined ROI mask, thereby accounting for the different sizes of each ROI. Information on the individual contribution of the nine ROIs, task-specific responses of individual ROIs and functional differences across the ROIs is blurred in this averaged measure. However, the advantage of the average working memory network measure lies in its ability to provide a simplified, sensitive, and normalized representation of overall network activity. This approach is particularly useful for high-level comparisons, reducing data complexity, and enhancing the robustness of findings. For details on the characteristics of the nine working memory ROIs see Abgottspon et al., 2022.

Preprocessing and quantification of ASL data were conducted with MATLAB R2019b (Mathworks, Natick, MA, USA), SPM, and FSL (FMRIB’s Software Library). Data preprocessing encompassed realignment, coregistration, segmentation, and normalization. Perfusion images were generated by performing pair-wise subtraction of label and control images. Quantification of CBF was performed using LOFT CBF toolbox. Regional CBF values in different cortical perfusion areas (anterior cerebral artery (ACA), middle cerebral artery (MCA), and posterior cerebral artery (PCA)) were extracted based on the Tatu perfusion territory atlas (Tatu et al., 1998). These territories were chosen based on previous cross-sectional analyses of this sample (Steiner et al., 2024). In addition, we calculated the global CBF as the average CBF across the entire brain mask.

### 2.6 Demographic characteristics and metabolic parameters

Age, sex, education, and intelligence quotient (IQ) were included as demographic characteristics. The highest level of education attained was categorized into three groups (high school, college / job training, and graduate school). IQ was assessed using a brief version of the WAIS-IV (van Ool et al., 2018, Petermann and Petermann, 2012). Plasma Phe, tyrosine, tryptophan as well as cerebral Phe levels were included as metabolic parameters. We included plasma tyrosine and tryptophan levels due to their critical roles as precursors for serotonin, dopamine, and noradrenalin synthesis. Their hypothesized lower levels in the brain are due to their competition with Phe to cross the blood–brain barrier (De Groot et al., 2010, Surtees and Blau, 2000). Plasma Phe, tyrosine, and tryptophan were obtained after an 8–12 h overnight fast with a high-performance ion-exchange liquid chromatography using a Biochrom 30 amino acid analyzer. To quantify cerebral Phe, proton magnetic resonance spectroscopy (^1^H-MRS) using a short-TE semi-LASER sequence was used. A large volume of interest of 50 × 75 × 20 mm^3^ (or reduced to 50 × 65 × 20 mm^3^ depending on head geometry) was semiautomatically placed in supraventricular white and gray matters with a small preponderance of WM (∼5 mm spacing to the roof of the lateral ventricles (Hoefemann et al., 2019)).

### 2.7 Statistical analysis

Categorical variables are presented as frequencies and percentages, and continuous variables as medians and interquartile ranges. In line with the analysis of the primary outcome of the present trial (Trepp et al., 2020), linear mixed models based on the restricted maximum likelihood estimation were used to assess the effect of the Phe intervention compared to the placebo intervention on neural activation, CBF, and task performance. This model accounts for repeated measures and enables the analysis of longitudinal changes throughout the study period. The dependent variables were the four-week values (neural activation, cerebral perfusion, and fMRI task performance) with fixed effects for intervention (Phe or placebo), baseline values, period (one or two), randomization stratification indicators (age, sex, study site), and a random intercept on participant ID. We report point estimates and two-sided 95 % confidence intervals (CI). For intervention (Phe or placebo), estimated marginal means with 95 % CI (adjusted for the mean of covariates) are reported for the dependent variables (West, 2009). To ensure that the Phe intervention was successful, linear mixed models were also calculated for metabolic parameters.

Predicted values were extracted from the linear mixed model to better disentangle the relationship between neural markers, cognition, and metabolic parameters after the Phe and placebo period. In a linear mixed model, predicted values refer to the values estimated for each observation based on the fixed and random effects (Bolker et al., 2009). We then separately calculated Spearman’s correlations with the predicted values of neural markers, cognition, and metabolic data for the Phe and placebo intervention. Neural activation in the working memory network and global CBF were used to reduce the number of variables. Statistical analyses were performed with SPSS version 29 and data visualization with the corrplot package in R (Wei et al., 2017). The significance level was set at *p* < 0.05. In addition, we present False Discovery Rate (FDR) correction to account for multiple comparisons. We used Spearman’s Roh as measure of effect size (*r*_s_ = 0.10 small effect, *r*_s_ = 0.30 medium effect, and *r*_s_ = 0.50 large effect (Cohen, 1988)).

In addition to the fMRI ROI analyses, exploratory whole-brain analyses were performed. A whole-brain one-sample *t*-test was conducted with the baseline data to ensure that our task activated working memory-related brain areas. For the exploratory whole-brain analyses, we used the contrast image from the first level analyses to calculate the difference (Δ) in neural activation between the post and pre-assessment for the Phe and placebo intervention (with the ImCal function in SPM). These Δ-contrast images were then used for second-level analyses. We computed a paired *t*-test with age as covariate to investigate whether there were differences in changes in neural activation between the placebo and the Phe intervention. The level of significance for all whole-brain analyses was set to *p* < 0.001 with a minimum extent threshold of 10 voxels (Lieberman and Cunningham, 2009). Family-wise error correction (FWE, *p* < 0.05) was applied at the cluster-level to correct for multiple comparisons. Voxel-wise whole-brain analyses were performed with SPM. The Automated Anatomical Labeling (aal) atlas was used to identify the corresponding neuroanatomical areas for the MNI coordinates (Tzourio-Mazoyer et al., 2002). Visualization of the MRI data was performed with MRIcroGL using the MNI 152 template (https://www.nitrc.org/projects/mricrogl).

## 3 Results

### 3.1 Participant characteristics

Of the 71 patients assessed for eligibility, 15 did not meet the inclusion criteria, and 26 declined to participate. Thirty patients were randomized to receive either Placebo-Phe or Phe-Placebo. Of the 30 patients randomized, 27 were included in the present study. One patient was excluded as they declined to participate after the first baseline assessment, and two participants were excluded due to movement artifacts. For three of the included 27 patients, neuroimaging data were not available at the third study visit (T3) due to technical problems with the MRI (*n* = 1) or due to home visits because of the COVID-19 pandemic resulting in missing neuroimaging data (*n* = 2). At the fourth study visit (T4), neuroimaging was not available for one patient who dropped out of the study due to discontinuation of safe contraception and for another patient who completed a home visit because of the COVID-19 pandemic. Therefore, data were available for *n* = 27 for T1 and T2, *n* = 24 for T3, and *n* = 25 for T4.

Demographic characteristics and metabolic parameters are presented in Table 1 and Supplementary Table 1. No significant differences in demographic characteristics and metabolic parameters were observed between the two groups (Phe-placebo or placebo-Phe). Plasma Phe and cerebral Phe were significantly increased after the Phe intervention (plasma Phe point estimate = 552 µmol/L, 95 % CI [421; 683], *p* < 0.001; cerebral Phe point estimate = 0.106 mmol/L, 95 % CI [0.083; 0.130], *p* < 0.001) and returned to baseline after termination of the Phe phase (see Fig. 4 in Trepp et al. (2024)). Similarly, a significant effect of the Phe intervention was found on plasma tyrosine (point estimate = 9 µmol/L, 95 % CI [2; 16], *p* = 0.012). Plasma tryptophan was not significantly higher after the Phe intervention compared to the placebo intervention (point estimate = −2 µmol/L, 95 % [−5; 1], *p* = 0.125).

**Table 1:** Demographic characteristics and metabolic parameters at baseline (T1).

	Overall (*n* = 27)	Phe-placebo (*n* = 13)	Placebo-Phe (*n* = 14)
Age − years	35.7 (29.1–38.3)	35.7 (29.3–38.2)	36.1 (21.0–43.4)
Sex, females (%)	12 (44.4)	6 (46.2)	6 (42.9)
Education			
High school	1 (3.7)	0	1 (7.1)
College/job training	22 (81.5)	12 (92.3)	10 (71.5)
Graduate school	4 (14.8)	1 (7.7)	3 (21.4)
IQ	97 (90–109)	100 (87–108)	96 (90–109)
Cerebral Phe (mmol/L)^1^	0.146 (0.137–0.191)	0.142 (0.137–0.192)	0.150 (0.132–0.179)
Plasma Phe (µmol/L)	749 (555–959)	733 (574–971)	751 (540–1009)
Plasma tyrosine (µmol/L)	38 (34–46)	44 (36–48)	37 (32–45)
Plasma tryptophan (µmol/L)	37 (30–40)	38 (37–42)	32 (29–39)

*Notes*. Categorical variables are presented in frequencies (percentages), continuous variables in median (interquartile range). ^1^Available for *n* = 26.

### 3.2 Neuroimaging data

**Fig. 1:**
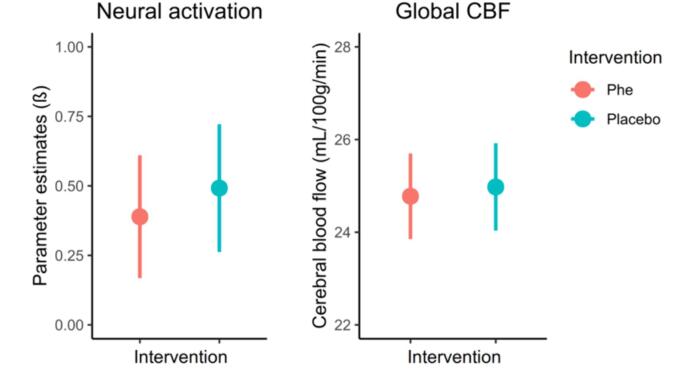
Neural activation in the working memory network and global cerebral blood flow after the Phe and placebo intervention. Displayed are estimated marginal means with associated 95 % confidence interval for the Phe and placebo intervention.

The results of the working memory fMRI data analyses are displayed in Fig. 1 and Table 2. Our analyses revealed no statistically significant effect of the Phe intervention compared to the placebo intervention on neural activation of the entire working memory network (point estimate = −0.10, 95 % CI [−0.38, 0.18], *p* = 0.455).

**Table 2:** Model estimates from the linear mixed model with the working memory fMRI ROIs based on the Neurosynth database as dependent variables.

Area	Hemisphere	Center of mass MNI coordinate			Placebo EMM [95 % CI]	Phe EMM [95 % CI]	Point Estimate [95 % CI]	*p*
		x	y	z				
Insula	L	−30	23	0	0.33 [0.12; 0.53]	0.22 [0.02; 0.42]	−0.11 [−0.35; 0.13]	0.362
Insula	R	33	22	0	0.28 [0.05; 0.51]	0.06 [−0.16; 0.28]	−0.22 [−0.52; 0.08]	0.140
Inferior frontal gyrus	R	48	8	25	0.36 [0.09; 0.63]	0.25 [−0.01; 0.50]	−0.11 [−0.47; 0.24]	0.529
Middle frontal gyrus	L	−36	52	10	0.29 [−0.02; 0.60]	0.33 [0.04; 0.63]	0.04 [−0.31; 0.39]	0.814
Middle frontal gyrus	L	−39	13	36	0.46 [0.26; 0.67]	0.39 [0.19; 0.60]	−0.07 [−0.30; 0.16]	0.537
Middle frontal gyrus	R	41	37	24	0.30 [0.03; 0.57]	0.14 [−0.12; 0.40]	−0.16 [−0.48; 17]	0.327
Superior frontal gyrus	R	16	10	51	0.36 [0.17; 0.56]	0.29 [0.10; 0.48]	−0.07 [−0.31; 0.17]	0.559
Inferior parietal lobule	L	−31	−57	45	0.72 [0.43; 1.00]	0.62 [0.35; 0.89]	−0.10 [−0.47; 0.27]	0.580
Inferior parietal lobule	R	32	−55	45	0.63 [0.32; 0.94]	0.49 [0.20; 0.78]	−0.14 [−0.53; 0.24]	0.455

*Notes*. CI = Confidence interval; EMM = Estimated marginal means; L = Left; *p* = Level of significance, uncorrected; R = Right.

A focused analysis with the nine working memory ROIs as dependent variable showed no significant effect of the Phe intervention compared to the placebo intervention on neural activation (Table 2). Despite the non-significant effect of the Phe intervention compared to the placebo intervention, it is noteworthy that there was a reduction of the mean neural activation across eight of the nine working memory ROIs. Neural activation was lower following the Phe intervention compared to the placebo intervention (see point estimates in Table 2). To exclude the possibility that these findings rely on the definition of the ROIs, we also created four ROIs based on the cerebral clusters associated with working memory performance found at T1 (Supplementary Fig. 1). In post-hoc analyses, parameter estimates were extracted for every timepoint, and the linear mixed model was applied. In line with the analyses of the nine working memory ROIs based on the Neurosynth database, no significant differences in neural activation were found after the Phe intervention compared to the placebo intervention (Supplementary Table 2).

Exploratory working memory fMRI whole-brain analyses did not reveal any differences in Δ neural activation between the Phe and placebo phase (all clusters *p*_FWE_ > 0.05, Supplementary Fig. 2). Of note, only participants with available fMRI data at all four timepoints were included in this analysis (*n* = 23).

The findings from the ASL data analyses are displayed in Fig. 1 and Table 3. No significant effect of the Phe intervention compared to the placebo intervention was found for global CBF. Similarly, the examination of CBF across the sixteen arterial vascular territories did not yield statistically significant differences among the intervention groups (*p* > 0.05). Again, it is noteworthy that the mean CBF was reduced in fifteen out of sixteen arterial vascular territories, even though the Phe intervention did not show a statistically significant effect compared to the placebo intervention (see point estimates in Table 3).

**Table 3:** Model estimates from the linear mixed model with CBF in arterial vascular territories as dependent variables.

Area		Placebo EMM [95 % CI]	Phe EMM [95 % CI]	Point Estimate [95 % CI]	*p*
Global CBF		24.98 [24.03; 25.92]	24.78 [23.85; 25.70]	−0.20 [−1.44; 1.04]	0.741
L ACA		21.80 [20.80; 22.81]	21.21 [20.22; 22.20]	−0.59 [−1.79; 0.61]	0.318
	L ACA anterior	23.21 [21.92; 24.51]	23.08 [21.81; 24.34]	−0.14 [−1.50; 1.23]	0.836

	L ACA posterior	30.16 [28.48; 31.83]	29.57 [27.91; 31.23]	−0.59 [−2.46; 1.28]	0.519
L MCA		22.23 [21.30; 23.16]	21.56 [20.65; 22.48]	−0.67 [−1.83; 0.49]	0.243
	L MCA anterior	20.89 [19.95; 21.83]	20.13 [19.22; 21.05]	−0.76 [−1.84; 0.33]	0.163
	L MCA middle	22.85 [21.79; 23.90]	22.40 [21.35; 23.45]	−0.44 [−1.67; 0.79]	0.459
	L MCA posterior	22.60 [21.22; 23.98]	21.34 [20.00; 22.68]	−1.26 [−2.91; 0.40]	0.129
L PCA		28.00 [26.56; 29.45]	27.36 [25.93; 28.79]	−0.64 [−2.19; 0.91]	0.399

R ACA		17.79 [16.71; 18.86]	17.43 [16.39; 18.48]	−0.35 [−1.56; 0.86]	0.555
	R ACA anterior	18.44 [17.25; 19.63]	18.02 [16.87; 19.17]	−0.42 [−1.69; 0.85]	0.498
	R ACA posterior	26.21 [24.52; 27.89]	26.16 [24.51; 27.81]	−0.05 [−2.04; 1.94]	0.961
R MCA		20.66 [19.81; 21.51]	19.96 [19.12; 20.80]	−0.70 [−1.85; 0.45]	0.224
	R MCA anterior	18.99 [18.08; 19.89]	18.09 [17.21; 18.98]	−0.89 [−2.11; 0.32]	0.145
	R MCA middle	22.12 [21.22; 23.03]	21.33 [20.44; 22.21]	−0.79 [−2.01; 0.42]	0.195
	R MCA posterior	19.08 [18.01; 20.15]	18.91 [17.87; 19.96]	−0.16 [−1.55; 1.22]	0.808
R PCA		22.43 [20.84; 24.02]	23.19 [21.63; 24.74]	0.76 [−1.17; 2.68]	0.423

*Notes*. ACA = Anterior cerebral artery; CBF = Cerebral blood flow (in mL/100 g/min); CI = Confidence interval; EMM = Estimated marginal means; L = Left; MCA = Middle cerebral artery; *p* = Level of significance, uncorrected; PCA = Posterior cerebral artery; R = Right.

### 3.3 fMRI task performance

**Fig. 2:**
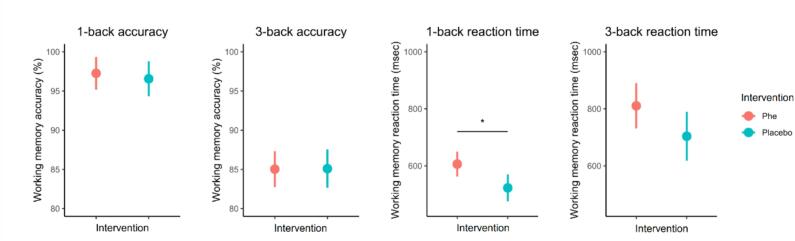
Working memory fMRI task performance after the Phe and placebo intervention. Displayed are estimated marginal means with associated 95 % confidence interval for the Phe and placebo intervention. Significant findings are marked with *.

Estimated marginal means of task performance during the working memory fMRI task for the Phe and placebo intervention are displayed in Fig. 2. Results of the linear mixed model analyses revealed no significant difference in working memory 1-back accuracy (point estimate = 0.70 %, 95 % CI [−1.74; 3.14], *p* = 0.560) or 3-back accuracy (point estimate = −0.06 %, 95 % CI [−3.25; 3.13], *p* = 0.970) for the Phe intervention compared to the placebo intervention. A significant effect of the Phe intervention on working memory 1-back reaction time was found (point estimate = 83.42 ms, 95 % CI [22.54; 144.30], *p* = 0.008), with slower 1-back reaction times during the working memory task after the Phe intervention. This significant result persisted following FDR correction (*p_FDR_* = 0.032). No significant difference in working memory 3-back reaction time was observed after the Phe intervention compared to the placebo intervention, although a trend towards slower reaction time after the Phe intervention was observed (point estimate = 106.78 ms, 95 % CI [−3.98; 217.55], *p* = 0.058).

### 3.4 Associations between neural markers (fMRI and CBF), cognition, and metabolic data

**Fig. 3:**
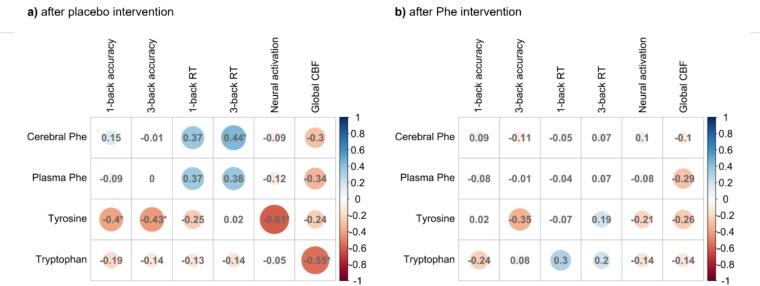
Correlations between predicted values of neural markers, cognition, and metabolic data a) after placebo intervention and b) after Phe intervention. Effect sizes with *r_s_* = 0.10 small effect, *r_s_* = 0.30 medium effect, and *r_s_* = 0.50 large effect. Effect sizes were rounded to two decimal places. Significant findings are marked with * (uncorrected *p*-values, two-sided). To reduce the number of variables, we used neural activation of the entire working memory network and not the individuals ROIs. Similarly, global CBF was used rather than CBF in the 16 arterial vascular territories.

Spearman’s correlation coefficients as a measure of effect sizes are plotted in Fig. 3. Overall, higher effect sizes were observed in the association between neural markers, cognition, and metabolic data following the placebo intervention compared to those observed after the Phe intervention.

With regard to neural markers and cognition, neural activation in the working memory network was significantly positively correlated with 1-back accuracy (*r_s_* = 0.604, *p* = 0.001) following the placebo intervention, which remained significant after FDR correction (*p_FDR_* = 0.008). No significant associations between neural markers − neither CBF nor fMRI metrics − and cognition were found after the Phe intervention.

In terms of neural markers and metabolic parameters, a significant positive correlation was found between neural activation in the working memory network and plasma tyrosine (*r_s_* = 0.609, *p* = 0.001) after the placebo intervention. Global CBF was significantly negatively associated with tryptophan (*r_s_* = −0.552, *p* = 0.004). Significant results persisted after FDR correction (*p_FDR_* = 0.008 and *p_FDR_* = 0.016, respectively). There were no significant correlations between neural markers and metabolic parameters after the Phe intervention.

Regarding metabolic parameters and cognition, cerebral Phe was significantly positively related to 3-back accuracy (*r_s_* = 0.443, *p* = 0.030) following the placebo intervention. Similarly, a significant negative association was found between plasma tyrosine and 1-back accuracy (*r_s_* = −0.389, *p* = 0.049) as well as 3-back accuracy (*r_s_* = −0.427, *p* = 0.033). None of the *p*-values remained significant after FDR correction (all *p_FDR_* > 0.05). No significant associations between metabolic parameters and cognition were found after the Phe intervention.

## 4 Discussion

In this double-blind, randomized, placebo-controlled, crossover, non-inferiority trial, a four-week period of oral Phe administration did not statistically significantly modulate working memory-related neural activation nor resting CBF in adults with early-treated classical PKU. Reaction time in the 1-back condition was significantly slower after the Phe intervention compared to the placebo intervention, whereas no significant effect of the intervention was found for accuracy. Relationships between neural markers, cognition, and metabolics differed following the Phe intervention compared to the placebo intervention.

Our ROI and exploratory whole-brain fMRI analyses suggest that a four-week high Phe intake simulating a controlled discontinuation of the Phe-restricted diet did not affect working memory-related neural activation significantly. This is the first study showing the effect of a four-week higher Phe level on fMRI outcomes. Sundermann et al. (2011) implemented a Stroop task during the fMRI acquisition and observed neural activation before and after a single administration with Phe. While the single Phe dosage was about 2–3 times higher than the daily dosage in the present study, we investigated the impact of Phe during a more extended period of four weeks. However, results from Sundermann et al. (2011) and our study point in the same direction and show that neither a one-day nor a four-week suspension of the Phe-restriction diet affects neural activation in a significant matter. However, we observed non-significantly decreased neural activation in eight out of nine working memory ROIs after the Phe intervention, indicating a certain pattern of neural activity change which did not reach significance in our study. Whether a long-term discontinuation (e.g., one year) of Phe-restriction might impact neural activation differently, remains to be established.

Similarly, no significant effect of the Phe intervention was found for CBF. Neither global CBF nor CBF in the 16 vascular territories statistically differed following the Phe or placebo intervention. Again, it is noteworthy that the mean CBF was not significantly reduced in fifteen out of sixteen vascular territories following the Phe intervention, suggesting a pattern of small changes in CBF after the Phe intervention. In a cross-sectional study including the present sample, Steiner et al. (2024) showed reduced CBF in the left anterior and middle MCA as well as the right posterior MCA in adults with PKU compared to controls. Together with the results of the present study, this suggests that CBF alterations occur in patients with PKU when comparing them to controls cross-sectionally. However, CBF alterations are unrelated to the concurrent Phe level in our longitudinal study design.

Contrary to our results, Muri et al. (2024) reported extensive significant declines in cortical gray matter accompanied by elevated white matter volume assessed with structural imaging following the four-week Phe intervention. These structural alterations may represent early adjustments or modifications that have not yet been fully detected in measurable changes in neural activity or CBF. It could be hypothesized that structural and functional neural changes do not necessarily occur in parallel but could also take place sequentially. Mapping structure–function relationships is complex and is influenced by several factors (Suárez et al., 2020). Thus, structural changes observed in the study by Muri et al. (2024) could precede functional changes. This further emphasizes the need for neuroimaging studies examining the impact of a high Phe load over a more extended period of time (e.g., one year). In addition, a high Phe level could indirectly influence brain structure through other mechanisms not directly detected by task-based fMRI or ASL, such as neuroinflammation, alterations in neurotransmitter levels, or white matter changes (Surtees and Blau, 2000, Bruinenberg et al., 2019, Anderson and Leuzzi, 2010). It has been hypothesized that neuroinflammatory processes occur in PKU, but more so in patients who received treatment later in life and were thus not early-treated (Ferreira et al., 2021). It may take longer for these effects to manifest as significant changes in neural activation or CBF.

Although we did not find an effect of the Phe intervention on a neural level, we observed slower reaction times in the 1-back condition after the Phe intervention compared to the placebo. This is in line with prior research indicating that adults who have discontinued the Phe-restricted diet exhibited slower reaction times, while stricter control of Phe levels resulted in a significant improvement in reaction times. However, reaction times were comparable between the on-diet and the control group (Dawson et al., 2011). Task accuracy was comparable after the Phe intervention compared to the placebo intervention. This is in line with the findings on the primary and secondary outcomes of this trial (Trepp et al., 2024). No significant effect of the Phe intervention was found for various cognitive functions such as working memory, cognitive flexibility, alertness, and divided attention. However, inhibition and sustained attention – outcomes with a speed or reaction time component – differed significantly between the Phe and placebo interventions. This implies that cognitive tasks relying on intact processing speed could be vulnerable to elevated Phe levels, while accuracy outcomes might be less affected by increased Phe, indicating a differential effect of high Phe on cognition.

Processing speed strongly relies on the integrity of the white matter (Penke et al., 2010, Turken et al., 2008). Cerebral white matter tracts form a complex network, allowing fast signal transmission between different brain areas (Penke et al., 2010, Turken et al., 2008). The speed of this signal transmission depends on the myelination of the white matter tracts, with higher myelination being associated with higher processing speed (Chopra et al., 2018). White matter integrity can be studied using diffusion tensor imaging (DTI). This approach analyses and visualizes the diffusion of water molecules along white matter tracts to investigate white matter integrity (Assaf and Pasternak, 2008). During the past decades, PKU has predominantly been described as a disease particularly affecting white matter integrity (Anderson and Leuzzi, 2010). DTI results of our earlier cross-sectional investigation, which included a similar patient sample, showed widespread alterations in posterior white matter tracts (Muri et al., 2023). These previous findings may help elucidate why we observed an impact of the Phe intervention on cognitive performance but did not detect a corresponding effect at the neural level. Performing a working memory task requires temporarily storing and processing information at a certain speed (Baddeley, 1983). Working memory performance is, therefore, tightly related to processing speed (Ackerman et al., 2002). Thus, the observed differences in reaction time could be driven by the integrity or the volume of the white matter rather than the neural activation assessed with fMRI.

We further examined associations between neural markers, cognition, and metabolic parameters following the Phe and the placebo intervention. There was a significant association with large effect size between neural activation and 1-back accuracy after the placebo intervention, which we did not see following the Phe intervention. Also, tyrosine and tryptophan were related to cognition and neural markers following the placebo intervention but not after the Phe intervention. Both levels remained within the reference ranges following the Phe and the Placebo intervention. It seems that exposure to high Phe somehow disrupts these associations. Whether and to what extent this is clinically relevant cannot be determined based on our data. Interestingly, tyrosine and tryptophan but not plasma Phe were associated with cognition or neural markers after the placebo intervention. This finding is comparable to the results of our cross-sectional study, where neural activation was associated with tyrosine and tryptophan rather than Phe (Abgottspon et al., 2022). Together with the findings from the cross-sectional study, where neural activation was related to tyrosine and tryptophan rather than Phe, this supports the notion that additional metabolic parameters play a pivotal role in the pathophysiology of PKU. Indeed, other metabolic parameters next to Phe levels, such as Phe-to-tyrosine ratio, Phe-variability, or cerebral tyrosine, have been linked to cognitive performance (Waisbren et al., 2016, Jahja et al., 2014, Hood et al., 2014, Romani et al., 2019).

This study further contributes to the discussion on safe Phe levels during adulthood. Our findings suggest that a high Phe exposure does not inherently have a detrimental impact on neural markers and cognition. Instead, it reflects differential effects depending on the variable being considered. We did not find any significant impact of the four-week Phe intervention on neural activation, CBF, and task accuracy, but 1-back reaction times were slower under high Phe. The life-long Phe-restricted diet comes with economic, psychological, and social challenges. Therefore, not only PKU-related symptoms but also dietary restrictions can affect health-related quality of life (Ford et al., 2018, MacDonald et al., 2010, Burgard et al., 2017, Olofsson et al., 2022, Bhashyam et al., 2019, Maissen-Abgottspon et al., 2023). Olofsson et al. (2022) suggest a trade-off between the benefits of strict dietary adherence and a more lenient diet. Strict dietary adherence might reduce PKU-related symptoms but increases dietary constraints, whereas a more lenient diet reduces the burden of the diet but takes into account possible PKU-related symptoms (Olofsson et al., 2022). Considering the patients' situation and needs, an individualized approach could be pursued instead of a target threshold (Burgard et al., 2017, Lachmann and Langeveld, 2024). Our results may be used to reassure patients, especially those with difficulties adhering to the Phe-restricted diet, that higher Phe during at least a limited period does not detrimentally influence their concurrent brain function in respect to the functional outcome measures used in the present study (Lachmann and Langeveld, 2024). However, slight non-significant reductions in mean neural activation and CBF after a high Phe load must be acknowledged.

The major strength of this study is the study design, which allows a causal conclusion to be drawn on the effect of a four-week period of oral Phe administration on neural markers and cognition. Also, the sample size is relatively large when considering the rarity of the disease; however, all findings must be interpreted with consideration to the sample size. Some limitations have to be acknowledged. First, owing to the design of the four-week discontinuation of the Phe-restricted diet, conclusions about long-term effects (e.g., one year) cannot be drawn. Second, the design of our fMRI task with the 1-back condition as the baseline condition and the 3-back condition as the working memory condition did not allow us to differentiate between different *n*-back loads. Future studies should incorporate multiple *n*-back loads (1-back to 3-back with 0-back as baseline). This approach would also allow the study of functional connectivity during task performance, investigating how spatially distinct areas in a functional network cooperate while performing a task (Huettel et al., 2014, Friston et al., 1997). Third, given our fMRI design, we focused on the neural basis of working memory. However, as our data suggests that cognitive domains with a processing speed component could be more sensitive to higher Phe, future task-based fMRI studies could focus on neural correlates of processing speed. Similarly, we used a combination of resting ASL with task-based fMRI in our study. Integrating both neuroimaging approaches enhances the study's neuroimaging scope, providing complementary insights into task-related neural activations and cerebral perfusion dynamics at rest. Future studies could further enrich our findings by incorporating a resting-state fMRI to better understand baseline brain function and its relationship with cognitive task performance. Fourth, our study utilized a relatively experimental setting. Further investigations with larger sample sizes, longer durations, and a stronger emphasis on patient-reported outcomes could enhance understanding of the effects of Phe variability in PKU management.

### 4.1 Conclusion

In conclusion, results from this present non-inferiority trial simulating a four-week suspension of the Phe-restricted diet showed that a high Phe load did not uniformly affect neural markers and cognition. While no significant changes were observed in neural activation, CBF, or working memory task accuracy, slower reaction times were evident in the 1-back condition following the Phe intervention. Our results could be used to reassure patients, particularly those with difficulties maintaining a Phe-restricted diet, that higher Phe levels over a limited period of time do not have a detrimental effect per se on their concurrent brain function, as measured by the outcome measures in the present study. To gain a more profound understanding of the effect of elevated Phe on brain function, future studies should incorporate a neuroimaging design associated with processing speed, focus on long-term effects beyond four weeks, and explore the potential impact of diverse metabolic parameters.

## Declaration of Generative AI and AI-assisted technologies in the writing process

During the preparation of this work, the authors used chatGPT to enhance language quality. After using this tool/service, the authors reviewed and edited the content as needed and take full responsibility for the content of the publication.

## Funding

This study was supported by the the Bangerter Rhyner Foundation (Switzerland), the Fondation Rolf Gaillard pour la recherche en endocrinologie, diabétologie et métabolisme (Switzerland), the Nutricia Metabolics Research Fund (Netherlands), the Swiss Foundation for Nutrition Research, the Swiss National Science Foundation (grant 192706 and 184453), the Vontobel Foundation (Switzerland), and a young investigator grant from the Inselspital Bern (CTU grant). The funding sources had no involvement in the design of the study or the collection, analysis, and interpretation of the data.

## CRediT authorship contribution statement

**Stephanie Maissen-Abgottspon:** Writing – review & editing, Writing – original draft, Visualization, Project administration, Investigation, Formal analysis, Data curation. **Leonie Steiner:** Writing – review & editing, Software, Methodology, Formal analysis. **Raphaela Muri:** Writing – review & editing, Project administration, Investigation, Funding acquisition, Data curation. **Dilmini Wijesinghe:** Writing – review & editing, Formal analysis, Data curation. **Kay Jann:** Writing – review & editing, Supervision, Formal analysis, Data curation. **Yosuke Morishima:** Writing – review & editing, Supervision, Formal analysis, Data curation. **Michel Hochuli:** Writing – review & editing, Supervision, Methodology, Conceptualization. **Roland Kreis:** Writing – review & editing, Software, Formal analysis, Data curation. **Roman Trepp:** Writing – review & editing, Supervision, Methodology, Funding acquisition, Conceptualization. **Regula Everts:** Writing – review & editing, Supervision, Methodology, Investigation, Funding acquisition, Conceptualization.

## Declaration of competing interest

The authors declare that they have no known competing financial interests or personal relationships that could have appeared to influence the work reported in this paper.

## Data Availability

Data will be made available on request.
