# Supplementary material for: Effect of a four-week oral Phe administration on neural activation and cerebral blood flow in adults with early-treated phenylketonuria
Source: Neuroimage Clin. 2024 Aug 14;43:103654. doi: 10.1016/j.nicl.2024.103654 (PMC11367652; doi:10.1016/j.nicl.2024.103654)
Supplement: Supplementary Data 1 [file mmc1.docx]

**Supplementary material for**

**Effect of a four-week oral Phe administration on neural activation and cerebral blood flow in adults with early-treated phenylketonuria**

Stephanie Maissen-Abgottspon, Leonie Steiner, Raphaela Muri, Dilmini Wijesinghe, Kay Jann, Yosuke Morishima, Michel Hochuli, Roland Kreis, Roman Trepp, Regula Everts

**This file includes:**

**Supplementary Figure 1 and 2**

**Supplementary Table 1 and 2**


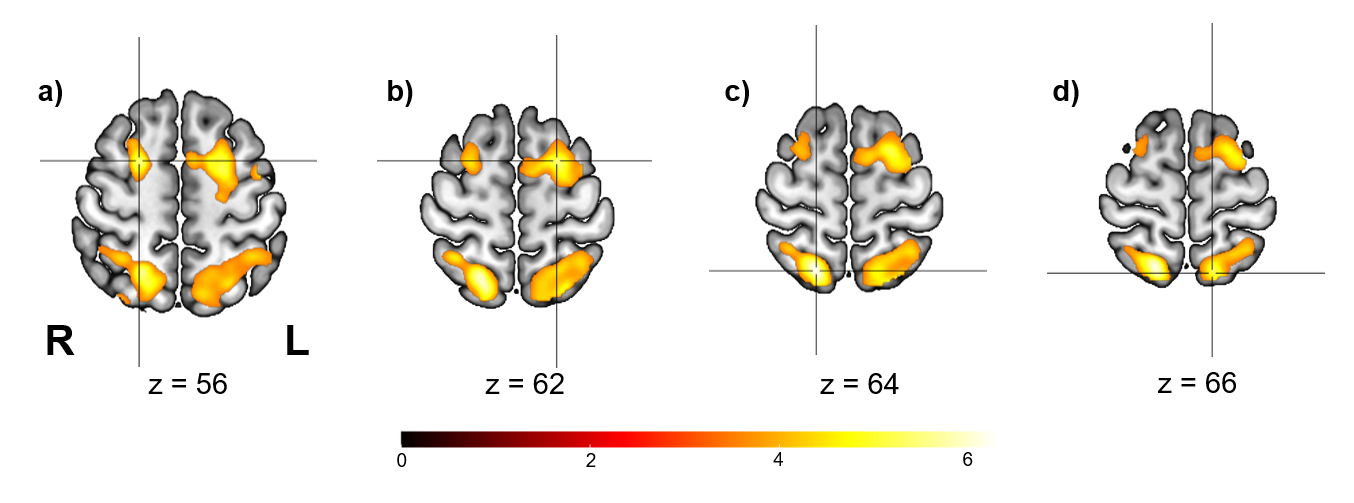


**Supplementary Figure 1**. **Working memory-related neural activation at baseline (*n* = 27)**. One-sample *t*-test of the baseline data revealed working memory-related neural activation in four cerebral clusters **a)** right superior frontal gyrus (x y z = [22 4 56], *k*_E_ = 404, *p*_FWE_ = .004); **b)** left superior frontal gyrus (x y z = [-22 4 62], *k*_E_ = 2106, *p*_FWE_ < .000); **c)** right superior parietal gyrus (x y z = [18 -62 64], *k*_E_ = 1847, *p*_FWE_ < .000); **d)** left superior parietal gyrus (x y z = [-14 -64 70], *k*_E_ = 1922, *p*_FWE_ < .000). The color bar refers to T-values and the z-coordinates to the MNI space. L = left, R = right.


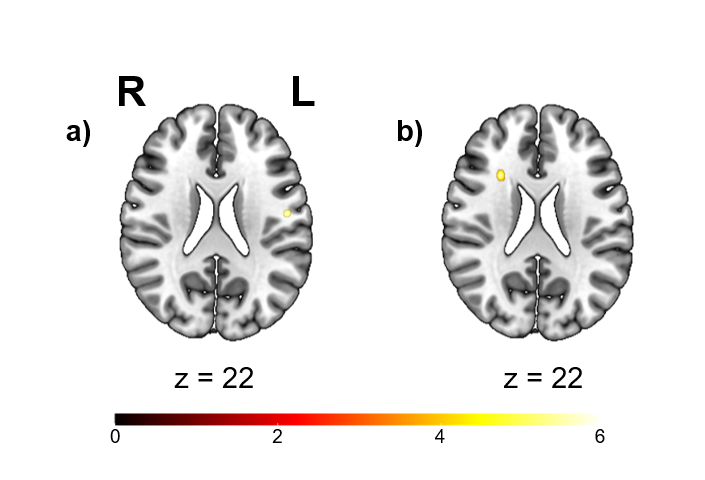


**Supplementary Figure 2. No significant changes in neural activation following the Phe and placebo intervention (*n* = 23).** Results of the whole-brain paired *t*-test. No significant clusters were observed for the contrast **a)** Delta Phe activation > Delta placebo activation (cluster in Figure not significant with *p*_FWE_ = .890); **b)** Delta placebo activation > Delta Phe activation (clusters in Figure not significant with *p*_FWE_ = .871 and *p*_FWE_ = .989 (not displayed in Figure)). Despite the high T-values indicated by the yellow clusters, the high *p*-values (*p*_FWE_ > 0.871) highlight that the difference in neural activation between the Phe and placebo intervention are likely due to chance. The color bar refers to T-values and the z-coordinates to the MNI space. L = left, R = right.

| **Supplementary Table 1**. Model estimates from the linear mixed model with metabolic variables as dependent variables. | | | | |
| --- | --- | --- | --- | --- |
| **Area** | **Placebo EMM**  [95 % CI] | **Phe EMM**  [95 % CI] | **Point Estimate**  [95 % CI] | ***p*** |
| Cerebral Phe (mmol/L) | .174  [.156; .193] | .281  [.264 ; .297] | .106  [.083; .130] | <.000 |
| Plasma Phe (µmol/l) | 893.30  [793.94; 992.67] | 1445.31  [1350.09; 1540.53] | 552.01  [421.28; 682.73] | <.000 |
| Plasma tyrosine (µmol/l) | 42.51  [37.28; 47.74] | 51.53  [46.49; 56.57] | 9.02  [2.13; 15.91] | .012 |
| Plasma tryptophan (µmol/l) | 36.14  [33.41; 38.87] | 33.83  [31.19; 36.47] | -2.31  [-5.30; 0.69] | .125 |
| *Notes*. Plasma Phe, tyrosine, and tryptophan are rounded to the next integer. EMM = Estimated marginal means; CI = Confidence interval; *p* = Level of significance, uncorrected. | | | | |

| **Supplementary Table 2.** Model estimates from the linear mixed model with the ROIs defined from the baseline activation as dependent variable. | | | | |
| --- | --- | --- | --- | --- |
| Area | **Placebo EMM**  [95 % CI] | **Phe EMM**  [95 % CI] | **Point Estimate**  [95 % CI] | ***p*** |
|  |  |  |  |  |
| Left superior frontal gyrus | .49 [.30; .69] | .40 [.22; .59] | -.09 [-.29; .11] | .364 |
| Right superior frontal gyrus | .49 [.26; .72] | .43 [.21; .65] | -.06 [-.35; .22] | .641 |
| Left superior parietal gyrus | .86 [.52; 1.20] | .76 [.44; 1.09] | -.10 [-.53; .34] | .648 |
| Right superior parietal gyrus | .67 [.38; .96] | .48 [.20; .76] | -.19 [-.53; .14] | .240 |
| *Notes*. EMM = Estimated marginal means; CI = Confidence interval; *p* = Level of significance, uncorrected. | | | | |
